# Breast Hemangioma with Slow Growth over 11 Years: A Case Report

**DOI:** 10.3390/reports9010023

**Published:** 2026-01-11

**Authors:** Anna Tabei, Tomoyuki Fujioka, Kazunori Kubota, Kumiko Hayashi, Tomoyuki Aruga, Iichiroh Onishi, Ukihide Tateishi

**Affiliations:** 1 Department of Diagnostic Radiology, Institute of Science Tokyo, Tokyo 113-8510, Japan; 2 Department of Radiology, Dokkyo Medical University Saitama Medical Center, Saitama 343-8555, Japan; 3 Department of Breast Surgery, Institute of Science Tokyo, Tokyo 113-8510, Japan; 4 Department of Pathology, Institute of Science Tokyo, Tokyo 113-8510, Japan

**Keywords:** breast hemangioma, breast angiosarcoma, breast cancer, ultrasound, magnetic resonance imaging

## Abstract

**Background and Clinical Significance**: Breast hemangioma is an extremely rare benign vascular tumor of the breast. Its imaging findings are nonspecific, and differentiation from malignant tumors such as encapsulated papillary carcinoma, mucinous carcinoma or angiosarcoma is often difficult. We report a case of breast hemangioma that showed slow growth over an 11-year period. **Case Presentation**: A woman in her 50s presented with a well-defined 11 mm mass in the upper outer quadrant of the left breast detected by ultrasonography. A core needle biopsy revealed a benign lesion, and follow-up was recommended. Eleven years later, the mass had increased to 27 mm. Magnetic resonance imaging showed high signal intensity on T2-weighted images and a fast-plateau enhancement pattern extending from the periphery to the center. Although malignancy was suspected, vacuum-assisted biopsy revealed a hemangioma. **Conclusions**: Breast hemangioma can show slow enlargement over a long period. Recognition of a characteristic peripheral-to-central enhancement pattern may aid in distinguishing this benign vascular lesion from malignant tumors.

## 1. Introduction and Clinical Significance

Breast hemangioma is a rare benign vascular tumor, accounting for approximately 0.4% of all breast tumors. Although reported in up to 11% of autopsy cases, clinically and radiologically diagnosed cases are extremely rare. Most lesions are asymptomatic and incidentally detected, typically smaller than 2.5 cm [1,2,3,4]. Because imaging findings are nonspecific, differentiation from fibroadenoma, mucinous carcinoma, encapsulated papillary carcinoma (EPC), and angiosarcoma is required. The pathogenesis of breast hemangioma remains unclear, but hormonal stimulation, trauma, and chronic inflammation have been proposed as potential contributing factors [5]. They usually remain stable over time or may even regress, but slow progressive enlargement can occasionally occur. In such cases, distinguishing them from malignant vascular or papillary tumors can be challenging on imaging alone.

From a clinical perspective, accurate recognition of breast hemangiomas is essential to avoid unnecessary surgical excision or overtreatment, especially when imaging features overlap with low-grade angiosarcoma or mucinous carcinoma. Recent advances in magnetic resonance imaging (MRI) have enabled the visualization of vascular morphology and hemodynamics, which can contribute to a more confident diagnosis.

Here, we report a rare case of breast hemangioma that slowly enlarged over 11 years.

## 2. Case Presentation

A postmenopausal woman in her 50s with no significant medical history was evaluated.

**Figure 1:**
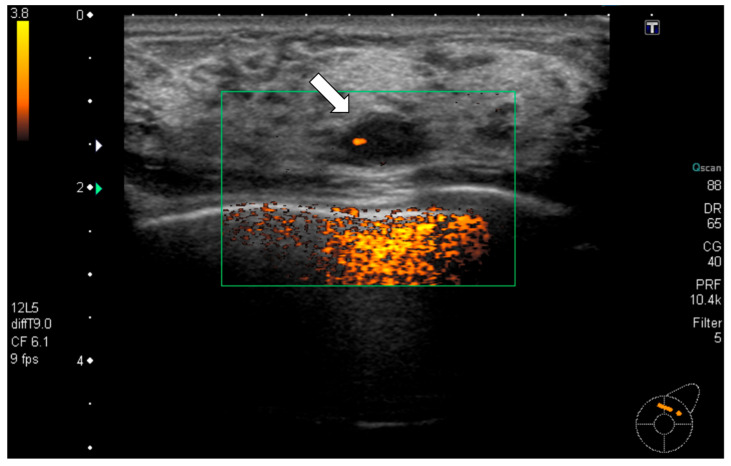
Ultrasonography (initial): A well-defined oval hypoechoic mass measuring 11 mm (arrow) was observed in the upper outer quadrant of the left breast with partial internal vascularity. Ultrasound-guided core needle biopsy was performed for this mass.

**Figure 2:**
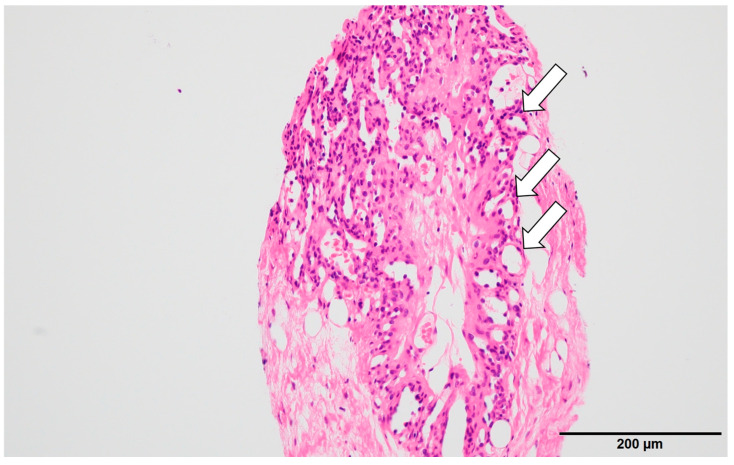
Histopathological findings (initial): Hematoxylin–eosin staining showed fibrous stroma with hyalinization containing small capillaries (arrow) and a few mammary ducts. No malignancy was found, and follow-up observation was recommended.

Radiological examination: Screening mammography showed architectural distortion in the central portion of the right breast. Ultrasound revealed a hypoechoic lesion corresponding to this area, diagnosed as a radial scar on core needle biopsy. In the upper outer quadrant of the left breast, an 11 mm oval, well-defined hypoechoic mass with partial blood flow was detected (Figure 1). Core biopsy showed fibrous stroma with capillary proliferation and no malignancy (Figure 2). Follow-up was recommended. However, during the subsequent 11-year interval, no follow-up examinations were performed because the patient discontinued hospital visits for personal reasons. As a result, regular clinical or radiological follow-up could not be conducted during this period.

**Figure 3:**
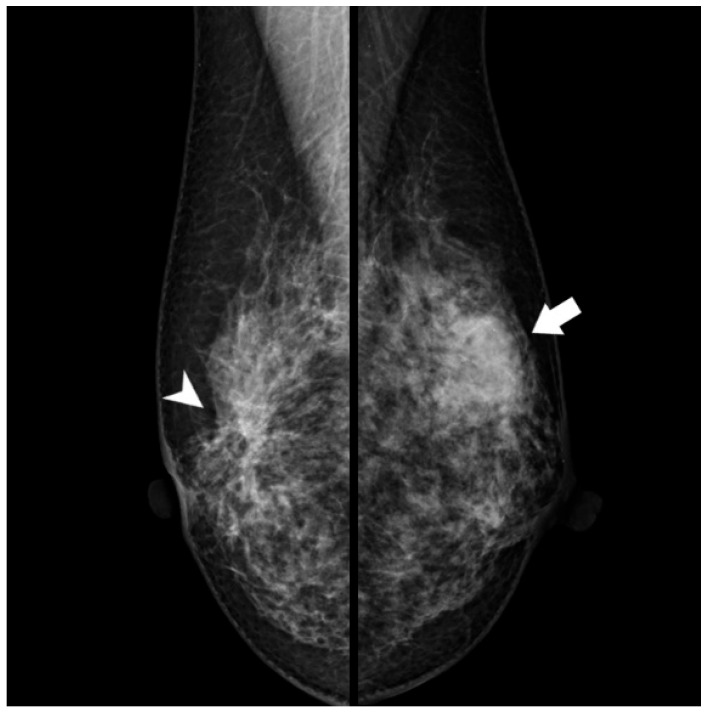
Mammography (after 11 years): A new dense, ill-defined mass was seen in the upper outer quadrant of the left breast (arrow). This mass was not observed 11 years earlier. Architectural distortion in the central portion of the right breast corresponding to a radial scar (arrowhead) remained unchanged.

**Figure 4:**
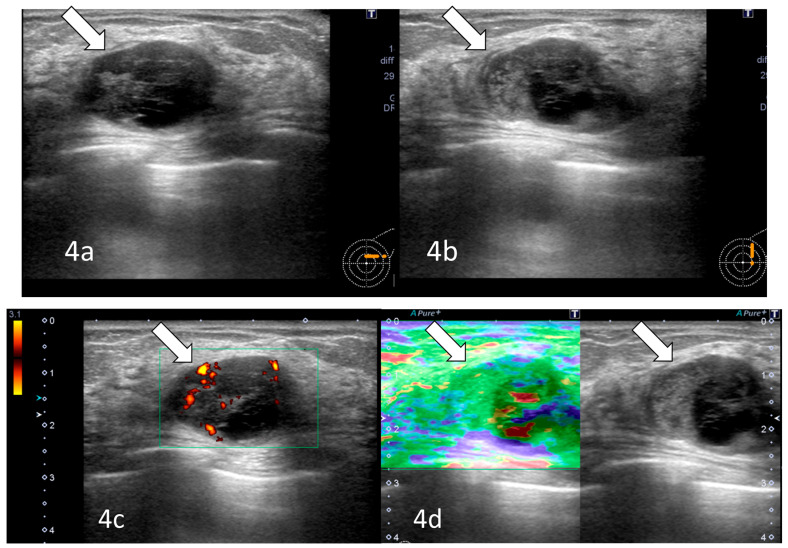
Ultrasonography (after 11 years): A 27 mm oval hypoechoic mass with central low and peripheral iso- to hyperechoic regions and posterior enhancement (arrows) was observed (**a**,**b**). Color Doppler imaging showed abundant peripheral flow (arrow) (**c**), and strain elastography showed mildly reduced strain (arrow) (**d**).

**Figure 5:**
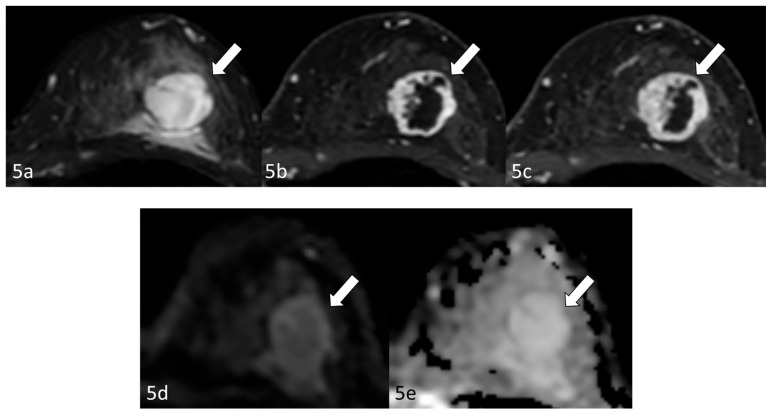
Magnetic resonance imaging (after 11 years): Magnetic resonance imaging (MRI) demonstrated a well-circumscribed round mass (arrow) with high signal intensity on fat-suppressed T2-weighted images (**a**). Dynamic contrast-enhanced imaging demonstrated spatially heterogeneous enhancement spreading from the periphery to the center, with a fast-plateau enhancement pattern in the peripheral portion and a medium persistent enhancement pattern in the central portion (**b**,**c**). The mass (arrows) showed high signal intensity on diffusion-weighted imaging and a high apparent diffusion coefficient value (**d**,**e**).

Radiological examination after 11 years: Screening mammography showed a newly visible dense, ill-defined mass in the upper outer quadrant of the left breast (Figure 3). Ultrasound demonstrated a 27 mm oval hypoechoic mass with central low and peripheral iso-to-hyperechoic areas and posterior enhancement (Figure 4a,b). Color Doppler imaging revealed abundant peripheral vascularity (Figure 4c). Strain elastography showed mildly reduced strain (Figure 4d). On MRI, a well-circumscribed round mass with high signal intensity was observed on fat-suppressed T2-weighted images (Figure 5a). Dynamic contrast-enhanced imaging demonstrated spatially heterogeneous enhancement spreading from the periphery to the center, with a fast-plateau enhancement pattern in the peripheral portion and a medium persistent enhancement pattern in the central portion (Figure 5b,c). The mass showed high signal intensity on diffusion-weighted imaging and high apparent diffusion coefficient (ADC) values (Figure 5d,e). The mass was classified as Breast Imaging Reporting and Data System category 4B, and ultrasound-guided vacuum-assisted biopsy was performed.

**Figure 6:**
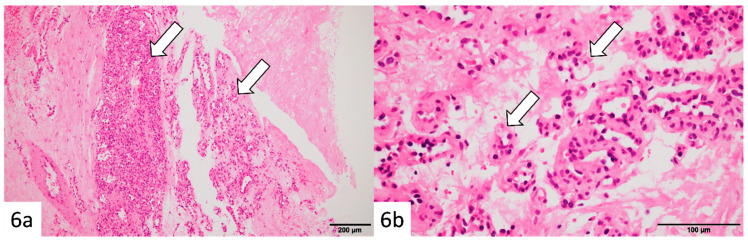
Histopathological findings (after 11 years): Hematoxylin–eosin staining of the vacuum-assisted biopsy specimen revealed clusters of small capillary-like vessels (arrows) with erythrocyte extravasation, fibrin deposition, and focal fibrosis (**a**,**b**). The endothelial cells exhibited no significant atypia. Similar findings were noted in the initial biopsy specimen, confirming a diagnosis of capillary hemangioma with gradual enlargement over 11 years.

Histopathology demonstrated proliferation of small capillary-like vessels with extravasation of red blood cells, fibrin deposition, and partial fibrosis (Figure 6a,b). Endothelial cells showed no significant atypia. The initial biopsy specimen also showed small vessels within fibrous stroma. A final diagnosis of capillary hemangioma with slow enlargement over 11 years was made.

## 3. Discussion

Breast hemangiomas are rare, and most are smaller than 2.5 cm, showing stability or regression over time. However, as in our case, some may demonstrate slow growth. Imaging findings are nonspecific: on mammography, they appear as well-defined masses; on ultrasound, as oval masses with variable echogenicity; and on MRI, as high T2 signal intensity masses with diverse enhancement patterns [1,2,3,4]. The most important differential diagnosis is angiosarcoma, a highly aggressive malignancy representing less than 0.04% of all breast cancers [6,7,8]. Angiosarcoma typically presents as a rapidly enlarging, ill-defined, heterogeneous mass often associated with skin discoloration or tenderness. In contrast, hemangiomas are slow-growing and well-circumscribed. On MRI, angiosarcoma demonstrates low-to-intermediate signal intensity on T1-weighted images and high-signal intensity on T2-weighted images. Variable kinetics have been described with low-grade angiosarcoma showing progressive enhancement and high-grade angiosarcomas showing washout-type enhancement with frequent visualization of large draining vessels [9].

In addition, EPC and mucinous carcinoma can also present with imaging features like those of hemangioma and therefore must be carefully differentiated [10,11,12].

The imaging appearance of mucinous carcinoma varies according to histologic subtype. Pure-type mucinous carcinoma, characterized by abundant mucin and low cellularity, typically shows mild diffusion restriction and relatively high ADC values, whereas mixed-type lesions, with larger solid components, tend to demonstrate stronger diffusion restriction and lower ADC values [10]. On dynamic contrast-enhanced MRI, pure-type lesions often display gradual enhancement (progressive or slow fill-in pattern) [11].

EPC is usually a well-circumscribed cystic mass containing internal papillary nodules. On MRI, it appears as a cystic lesion with internal enhancing solid nodules, shows high T2 signal intensity, and the papillary components typically exhibit early enhancement followed by a washout kinetic curve. In addition, EPCs often possess a capsule-like rim with a sharp interface between the lesion and the surrounding parenchyma [12].

In our case, MRI demonstrated heterogeneous enhancement kinetics within the lesion: the peripheral portion showed a fast initial enhancement followed by a plateau, whereas the central portion exhibited a more gradual and persistent enhancement. Spatially, the enhancement progressed from the periphery toward the center over time, resulting in a pattern resembling the peripheral nodular enhancement with centripetal fill-in characteristic of hepatic hemangiomas [13,14]. This distinction between temporal enhancement kinetics and spatial enhancement progression suggests that similar hemodynamic mechanisms may occur in breast hemangiomas. Recognition of this combined temporal and spatial enhancement pattern may therefore provide an important diagnostic clue indicating a benign vascular lesion.

From a pathological perspective, slow enlargement of a hemangioma does not necessarily indicate active cellular proliferation of the lesion itself, but rather may reflect secondary changes such as fibrosis, thrombosis, or organization after hemorrhage. These chronic alterations may account for the internal echogenic heterogeneity observed on ultrasonography and the mild stiffness detected on elastography. Therefore, if imaging and histopathological findings remain within the benign spectrum, a gradual increase in size alone should not be interpreted as malignant transformation.

## 4. Conclusions

Breast hemangioma is a rare benign tumor that can show gradual enlargement over time. Although differentiation from malignancy based solely on imaging can be difficult, the observation of peripheral-to-central enhancement may help suggest a benign vascular nature. Importantly, slow enlargement does not necessarily indicate neoplastic proliferation but may instead reflect secondary pathological changes, such as fibrosis or thrombosis within the lesion. This case illustrates both the imaging diversity and the long-term benign course of breast hemangioma.

## Data Availability

The original data presented in the study are included in the article, further inquiries can be directed to the corresponding author.
